# Time‐dependent systolic blood pressure within 72 h after endovascular treatment in large vessel occlusion stroke

**DOI:** 10.1002/brb3.3442

**Published:** 2024-03-07

**Authors:** Xin Jiang, Jinghuan Fang, Lijie Gao, Shuju Dong, Jian Wang, Fayun Hu, Li He

**Affiliations:** ^1^ Department of Neurology West China Hospital, Sichuan University Chengdu Sichuan China

**Keywords:** blood pressure, clinical outcome, endovascular treatment, large vessel occlusion stroke, time dependency

## Abstract

**Background:**

The association of systolic blood pressure (SBP) and ischemic stroke outcome has recently been proved to be varied at different time points within 72 h after acute ischemic stroke onset; however, the specific status of how SBP affects prognosis at different time points within 72 h after endovascular treatment (EVT) among patients with large vessel occlusion (LVO) remains unclear.

**Methods:**

Consecutive LVO patients treated with EVT were enrolled in our study. BP data were collected at eight time points (1, 2, 4, 8, 16, 24, 48, and 72 h post‐EVT). Outcome measure of interest was functional dependence, which was defined as mRS >2 at 90 days.

**Results:**

A total of 406 LVO patients treated with EVT from 2016 to 2022 were included. At 16 h after EVT, the relationship between SBP and functional dependence showed a nonlinear association. At other time points after EVT, SBP had linear relationships with functional dependence. Furthermore, higher SBP, as either a linear or quadratic term, had an adverse effect on functional outcome. In addition, three SBP trajectories were observed, and the high‐to‐low group was independently associated with functional dependence.

**Conclusion:**

Taken together, higher SBP within the first 72 h after EVT has a time‐dependent association with adverse clinical outcomes. Optimal blood pressure management during the first 72 h after EVT may be important to improve clinical outcome.

## Graphical abstract

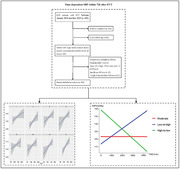

## 1 INTRODUCTION

Acute ischemic stroke (AIS) is the leading cause of disability and death in adults in China (Wu et al., 2019). Over 30% of patients with AIS experience large vessel occlusion (LVO) stroke, accounting for 61% of all stroke mortality and severe disability (Chen et al., 2015; Liu et al., 2011; Malhotra et al., 2017). Occlusion of an artery supplying the brain is the cause of LVO, which can lead to the death of brain tissue and focal neurological deficits (Prabhakaran et al., 2015). The treatment of LVO stroke has been transformed by the widespread adoption of endovascular treatment (EVT) since the publication of several landmark randomized clinical trials (RCTs) in the last decade (Goyal et al., 2015, 2016; Jadhav et al., 2021). However, even with successful recanalization after EVT, 54% of patients with LVO still have poor functional outcomes and do not regain functional independence (Goyal et al., 2016).

Blood pressure (BP) management after AIS is a proposed strategy to improve clinical outcomes, especially in those who receive reperfusion therapy (Goyal et al., 2017). Previous observational studies showed that higher mean systolic blood pressure (SBP) levels and BP variability within 72 h after the onset of AIS were independently associated with worse functional outcomes and higher mortality (Anadani et al., 2020; Katsanos et al., 2022; Manning et al., 2015). Moreover, several observational studies have proven that lower SBP goal after EVT will improve the clinical prognosis of patients with LVO (Anadani et al., 2020). Conversely, the ENCHANTED2/MT trial showed that the most appropriate management of BP after EVT remained controversial because its findings revealed that more intensive BP lowering (SBP target <120 mmHg) was harmful (Yang et al., 2022). In addition, Shin et al. (2020) found that the relationship between SBP and outcome varied at different time points within 72 h after AIS onset. Nevertheless, only 20% of the included patients in Shin's study received EVT, and there was no subgroup analysis in patients with LVO treated with EVT. Therefore, whether there is an association between SBP and prognosis at different time points within 72 h after EVT among patients with LVO remains unclear due to the lack of clinical evidence.

In the present study, we retrospectively investigated the association between BP values and clinical outcome at eight time points (1, 2, 4, 8, 16, 24, 48, and 72 h) within 72 h after EVT in patients with LVO, characterizing BP trajectories in the first 72 h after EVT to identify the relationship between BP patterns and clinical outcome.

## 2 MATERIALS AND METHODS

### 2.1 Study participants

All consecutive nonselected patients who were treated with EVT between January 2016 and June 2022 in West China Hospital were retrospectively reviewed. In general, the criteria for selecting patients were as follows: (1) aged 18 years or over; (2) a causative LVO in the anterior circulation; (3) time from stroke onset to groin puncture within 24 h; and (4) received EVT. We excluded patients who had any 1 or more of the following: (1) a known prestroke mRS ≥2; (2) insufficient BP data; (3) signs of a large infarct core prior to EVT; and (4) lost to follow‐up at 3 months. The EVT techniques used included direct aspiration, stent retriever, balloon dilation, and stent placement, and the best combination of these approaches was determined by the operator based on established clinical guidelines. This study was performed in accordance with the ethical principles of the 1964 Declaration of Helsinki and its later amendments and approved by the Ethics Committee of West China Hospital [No. 2020(69)]. The need to obtain written informed consent was waived because of the retrospective and observational nature of the study.

### 2.2 Data collection

Individual patient data with baseline characteristics and clinical information, including age, sex, history of hypertension, admission National Institutes of Health Stroke Scale (NIHSS), treatment with intravenous thrombolysis, the site of intracranial vessel occlusion, time to groin puncture, and thrombolysis in cerebral infarction score, were collected (Hirsch et al., 2016; Liebeskind et al., 2019).

BP was regularly measured by professional nurses with automated oscillometric devices (brachial cuff) on the nonparalytic arm and recorded into the electronic record system. BP parameters were routinely measured and recorded every 15 min for 2 h, every 30 min at 2–6 h, and every 2 h within 72 h after EVT. We extracted BP data at eight time points (1, 2, 4, 8, 16, 24, 48, and 72 h) after EVT (Shin et al., 2020, 2022).

### 2.3 Outcome measures

We used the 3‐month mRS score to assess the clinical outcome of patients with LVO, and functional dependence was defined as a 3‐month mRS score of 3–6. BP data were divided into eight categories to display the distributions for different BP levels at each time point. For our primary analysis, we compared linear and quadratic regression models analyzing the relationship between BP and 3‐month functional dependence at each time point.

### 2.4 Statistical analysis

The baseline characteristics of patients are presented as numbers of patients, percentages, means with standard deviations (SDs), and medians with interquartile ranges. The Shapiro–Wilk test was used to test for normality. All continuous variables are sufficiently normally distributed and presented as mean  ±  SD. The Student *t*‐test and the chi‐square test were performed to assess differences in clinical characteristics for continuous variables and categorical variables, respectively. The relationship between BP and outcome was assessed using adjusted models, and the predetermined covariates were as follows: age, sex, initial NIHSS score, stroke subtype, medical history, intravenous thrombolysis, onset to groin puncture, and successful recanalization. For the primary analysis, multivariable logistic regression models were constructed of SBP and diastolic blood pressure (DBP) measured at each time point. For all analyses, the subject's BP was used as a continuous variable. Due to the possibility of a nonlinear relationship between BP and outcome, both the linear and quadratic models were investigated. An Akaike information criterion (AIC) value and a likelihood ratio (LR) statistic were used to compare those two models (Aho et al., 2014; Bagherzadeh‐Khiabani et al., 2016).

By using latent variable mixture modeling, heterogeneous longitudinal data were divided into groups with similar patterns, and then the BP trajectories were generated (Nagin & Odgers, 2010; Nguena Nguefack et al., 2020). We described each trajectory according to their appearances. All statistical analyses were performed using R Studio, and all *p* values reported are two‐tailed. A *p* value of <.05 was considered significant.

## 3 RESULTS

### 3.1 Demographic and clinical characteristics

**FIGURE 1:**
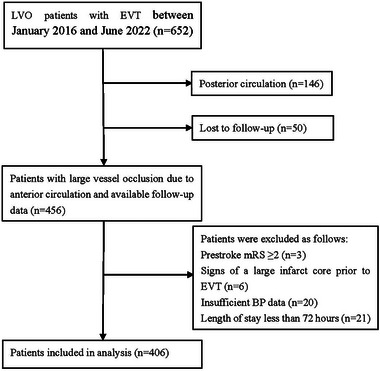
Flowchart of patient enrollment.

A total of 406 patients who received EVT with 48,086 BP measurements were included in this analysis (Figure 1; Table 1). The median age of all the patients was 70 years, and the median baseline NIHSS score was 15. Among all the included patients, 56.4% were men, 51% were diagnosed with hypertension, and 25.1% received intravenous thrombolysis. Moreover, 65% (*N* = 264) of the 406 patients had a 3‐month mRS score of 3–6 (functional dependence). The distributions with mean values and SDs of SBP and DBP at each time point after EVT are presented in Table S1. At 1 h after EVT, the mean SBP was 129.7 mmHg, and the mean DBP was 74.1 mmHg. One hour after EVT, BP remained relatively stable for the next seven time points (2, 4, 8, 16, 24, 48, and 72 h). The distributions of SBP at each time point are shown in Figure S1, and the distributions of DBP are shown in Figure S2. The distribution of functional dependence (mRS > 2) for different SBP and DBP groups at each time point after EVT is shown in Figures S3 and S4.

**TABLE 1:** Baseline and demographic characteristics, and univariate outcome results divided by functional outcome.

Characteristics	Overall	mRS 0–2	mRS 3–6	*p*
	*N* = 406	*N* = 142	*N* = 264	
Age, median	70.0 (57.0, 78.0)	64.0 (54.0, 73.0)	74.0 (61.0, 81.0)	<.001^*^
Male, *n* (%)	229 (56.4)	94 (66.2)	135 (51.1)	.005^*^
Hypertension, *n* (%)	207 (51.0)	66 (46.5)	141 (53.4)	.219
Diabetes mellitus, *n* (%)	70 (17.2)	20 (14.1)	50 (18.9)	.273
Smoking, *n* (%)	129 (31.8)	60 (42.3)	69 (26.1)	.001^*^
AF, *n* (%)	191 (47.0)	49 (34.5)	142 (53.8)	<.001^*^
Initial SBP	144.1 (24.8)	140.5 (21.0)	146.0 (26.5)	.035^*^
Initial DBP	86.1 (18.8)	87.1 (15.7)	85.5 (20.3)	.423
Admission NIHSS score	15 (11–18)	13 (9–16)	15 (12–19)	<.001^*^
Side (%)				.811
Left, *n* (%)	204 (50.2)	73 (51.4)	131 (49.6)	
Right, *n* (%)	202 (49.8)	69 (48.6)	133 (50.4)	
ICA, *n* (%)	168 (41.4)	48 (33.8)	120 (45.5)	.03^*^
M1, *n* (%)	174 (42.9)	66 (46.5)	108 (40.9)	.329
M2, *n* (%)	62 (15.3)	24 (16.9)	38 (14.4)	.599
TOAST (%)				.834
LAA	136 (33.5)	49 (34.5)	87 (33.0)	
CE	231 (56.9)	77 (54.2)	154 (58.3)	
Others	36 (9.6)	16 (11.3)	23 (8.7)	
Prior IVT, *n* (%)	102 (25.1)	42 (29.6)	60 (22.7)	.162
Onset to groin (min)	294 (227.8–386.0)	295 (224.5–408.5)	292 (230.0–364.0)	.603
Stent retriever, *n* (%)	293 (72.2)	92 (64.8)	201 (76.1)	.021^*^
Aspiration, *n* (%)	202 (49.8)	75 (52.8)	127 (48.1)	.423
Recanalization, *n* (%)	346 (85.2%)	137 (96.5%)	209 (79.2%)	<.001^*^
NIHSS, 24 h	14 (8–20)	7 (4–12)	18 (12.0–24.2)	<.001^*^
NIHSS, discharge	10 (4–20)	3 (1–6)	16 (10–30)	<.001^*^

Abbreviations: AF, atrial fibrillation; CE, cardiac embolism; DBP, diastolic blood pressure; ICA, internal carotid artery; IVT, intravenous thrombolysis; LAA, large‐artery atherosclerosis; M1, first segment of middle cerebral artery; M2, second segment of middle cerebral artery; mRS, modified Rankin scale; NIHSS, National Institutes of Health Stroke Scale; SBP, systolic blood pressure; TOAST, trial of org 10172 in acute stroke treatment.

* *p* < .05, significant in univariate analysis.

**FIGURE 2:**
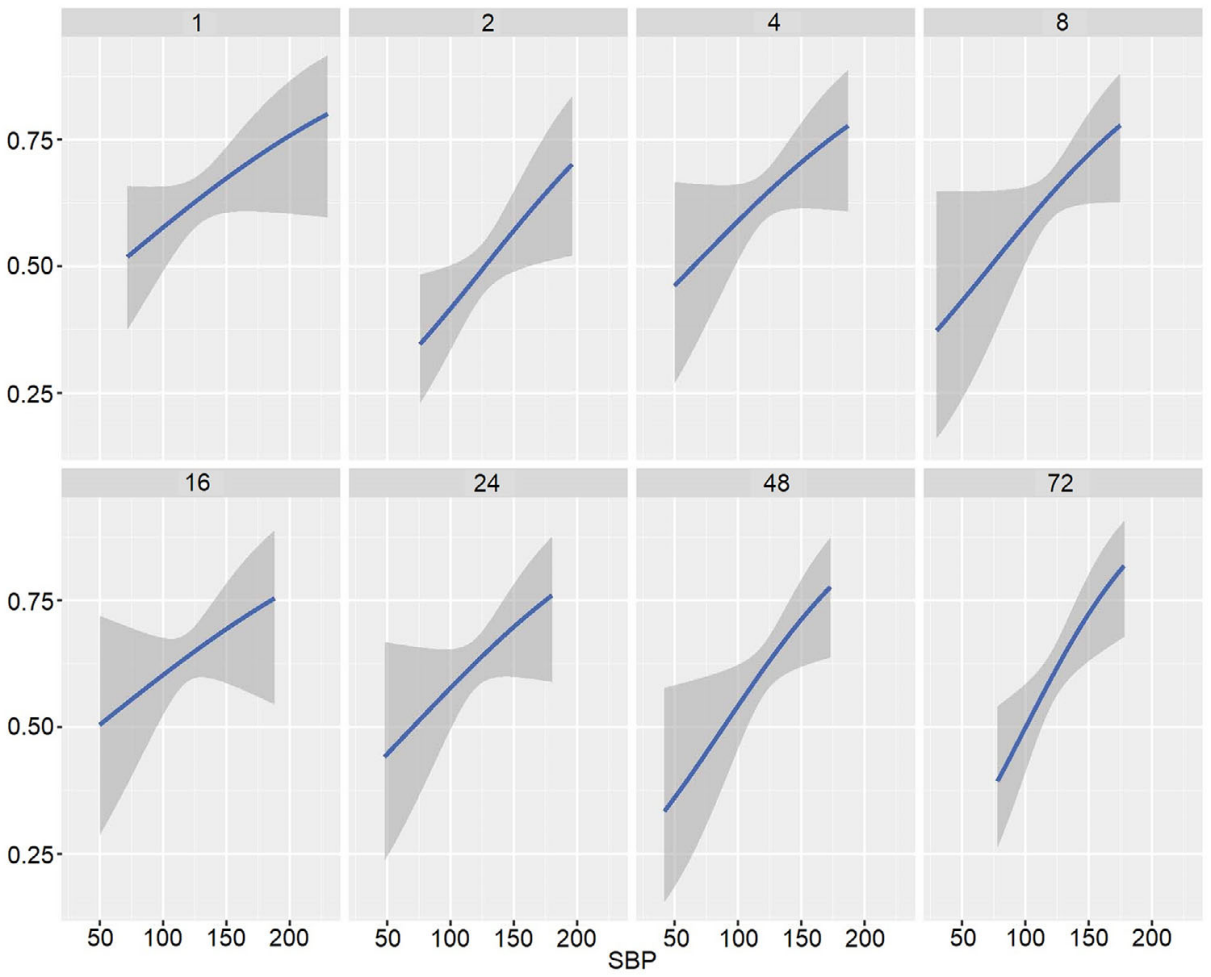
Probability of functional dependence (3‐month mRS 3–6) predicted by systolic blood pressure (SBP) at each time point.

Overall, functional dependence was more common in patients with higher SBP categories through eight time points. The probability of functional dependence (mRS 3–6) according to the SBP values was calculated using the adjusted models and displayed by the time points after EVT (Figure 2). In general, higher SBP was associated with worse outcome irrespective of different time points after EVT. For DBP, even though the slope (DBP/outcome) was discrepant at each time point (Figure S5), elevated DBP levels were related to worse outcomes, with the exception of 24 h after EVT.

### 3.2 Association between BP parameters and functional dependence

We used adjusted nonlinear and linear models to examine the relationship between BP and functional dependence at each time point after EVT. AIC differences between linear and quadratic models, the significance of the LR test, and the significance of SBP and DBP terms in the logistic regression models at each time point are presented in Table 2 after adjusting predetermined covariates of age, sex, initial NIHSS score, stroke subtype, medical history, intravenous thrombolysis, onset to groin puncture, and successful recanalization. Both the linear and nonlinear SBP terms were not statistically significant at 1, 2, 4, 8, 24, and 48 h, whereas the linear was statistically significant at 72 h. At 16 h, the nonlinear SBP term was statistically significant compared to the linear SBP term. For DBP, both the linear and nonlinear effects were not statistically significant at any of the eight time points after EVT.

**TABLE 2:** Comparisons of linear and quadratic regression models analyzing relationship between blood pressure (BP) and 3‐month functional outcome at each time point.

		1‐h	2‐h	4‐h	8‐h	16‐h	24‐h	48‐h	72‐h
	*p* of *β* ^2^								
**Linear model**	SBP	.549	.388	.546	.355	.335	.514	.271	.039
**Quadratic model**	SBP	.829	.768	.383	.799	.016	.689	.703	.637
	SBP^2^	.883	.694	.419	.901	.022	.635	.629	.506
**Model comparison**									
△AIC (L–Q)^a^		.007	.205	.579	.003	4.404	.313	.329	.566
*p* of △‐2LL^b^		.883	.691	.412	.899	.029	.622	.621	.503
	*p* of *β* ^2^								
**Linear model**	DBP	.729	.992	.909	.887	.414	.829	.353	.723
**Quadratic model**	DBP	.261	.219	.169	.655	.136	.801	.717	.542
	DBP^2^	.274	.216	.159	.666	.143	.777	.632	.567
**Model comparison**									
△AIC (L–Q)^a^		.999	1.415	1.843	.256	1.509	.036	.315	.219
*p* of △‐2LL^b^		.278	.204	.149	.664	.147	.778	.631	.567

*Note*: Variables adjusted were age, sex, initial NIHSS score, stroke subtype, medical history, smoking, intravenous rt‐PA use, onset to groin puncture, and successful recanalization.

Abbreviations: AIC, Akaike information criterion; DBP, diastolic blood pressure; SBP, systolic blood pressure.

^a^ A lower Akaike information criterion (AIC) value indicates a better model fit. L indicates linear and Q indicates quadratic.

^b^ If *p* of △‐2LL is under .05, quadratic model is significantly better in model fit.

### 3.3 BP trajectories

**FIGURE 3:**
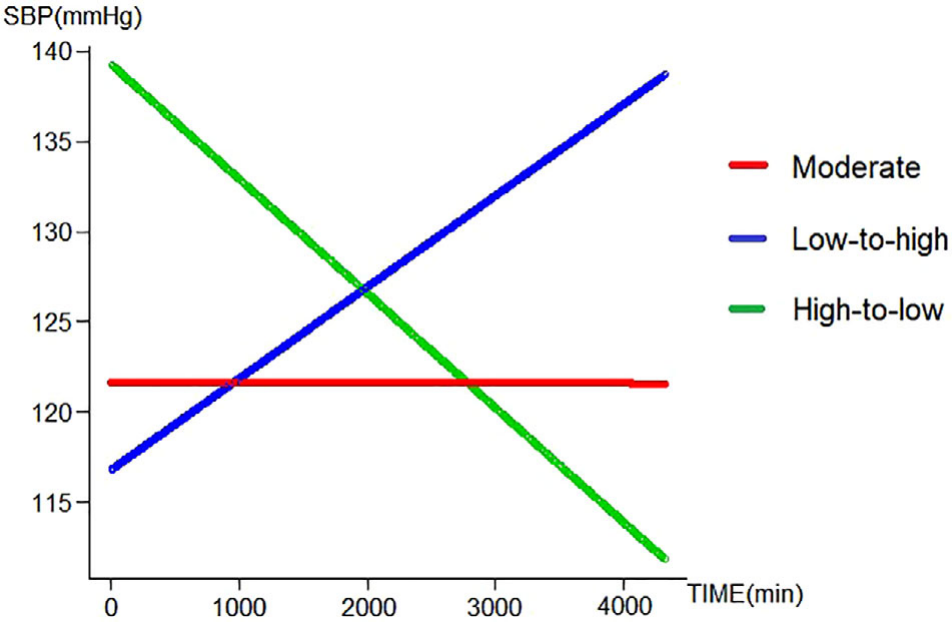
Systolic blood pressure (SBP) trajectories within 72 h after endovascular treatment (EVT).

Trajectory analysis generated three different SBP trajectories: moderate, low‐to‐high, and high‐to‐low (Figure 3). Compared with the moderate group, subjects in the high‐to‐low (adjusted OR, 3.88 [95% CI, 1.67–9.02]; *p* = .0016) trajectory groups had a significantly increased risk of functional dependence at 90 days (Table S2).

## 4 DISCUSSION

This retrospective study revealed that the relationship between BP and clinical outcome varies at different time points within 72 h after EVT. It demonstrated that higher SBP, as either a linear or quadratic term, had an adverse effect on clinical outcome.

Some studies have shown that LVO stroke has an ischemic penumbra with impaired autoregulation that is more sensitive to BP level changes (Jusufovic et al., 2015). Theoretically, successful recanalization could facilitate rapid flow changes in the ischemic vascular bed and lead to cerebral hyperperfusion syndrome, which may cause further brain injury, such as edema and hemorrhage (Khatri et al., 2012; Mizuma et al., 2018). The role of BP management in patients with LVO stroke receiving reperfusion therapy could be crucial, especially in those who received EVT (Mizuma et al., 2018).

BP levels usually increase at the acute phase of AIS, and evidence regarding early intervention for elevated BP levels in patients with LVO after EVT remains controversial, with no unequivocal consensus regarding the optimal BP target in LVO patients after EVT. Although the 2019 American Heart Association/American Stroke Association guidelines recommended maintaining an SBP ˂180/105 mmHg after EVT (Powers et al., 2019), randomized trials are still needed to substantiate this proposal. The BP TARGET study, which evaluated the impact of intensive SBP reduction on outcomes after successful reperfusion, suggested no benefit from intensive SBP goals in the first 24 h after EVT (Mazighi et al., 2021). Inconsistent findings were observed in the Enhanced Control of Hypertension and Thrombectomy Stroke Study (ENCHANTED2/MT), which compared more intensive treatment (SBP target <120 mmHg) with less intensive treatment (SBP target 140–180 mmHg) within 72 h after successful mechanical thrombectomy (Yang et al., 2022). The findings of ENCHANTED2/MT trial that included 816 patients who received successful mechanical thrombectomy showed that poor functional outcome was more common in the more intensive treatment group than the less intensive treatment group.

Many observational studies have shown that elevated BP during the acute phase after stroke onset is associated with poor outcome (Berge et al., 2015; Ishitsuka et al., 2014). A 2022 meta‐analysis of individual BP data after EVT concluded that increased mean systolic BP levels within 24 h after EVT were independently associated with a worse 3‐month functional outcome (Katsanos et al., 2022). Our study demonstrated that elevated SBP during the first 72 h after EVT in LVO stroke patients seemed to have an adverse impact on clinical outcome, which was consistent with previous observational findings but inconsistent with ENCHANTED2/MT trial. Some reasons may explain why the findings of ENCHANTED2/MT differed from current observational studies. First, the majority of enrolled patients (52%) in ENCHANTED2/MT trial had large artery atherosclerosis, which differs from our study population (33.5%). Second, all included patients in ENCHANTED2/MT trial had elevated BP (≥2 successive measurements of SBP ≥140 mmHg for >10 min) within 3 h of successful reperfusion. In the real‐world practice, low SBP could be observed after EVT in some patients who might be considered a physiological response to better reperfusion and cerebral autoregulation. At last, the participants in ENCHANTED2/MT trial had a large mismatch of perfusion deficit and achieved successful recanalization, patients who were unlikely to benefit from EVT and those with incomplete recanalization were underpowered to draw conclusions. To the best of our knowledge, some neurologists and neurointerventionalists in China and other countries have already adopted an SBP target of less than 140 mmHg or even less than 120 mmHg in routine practice (Yang et al., 2022). BP management after EVT should be individualized according to many factors, such as stroke subtypes, recanalization status, previous history of hypertension, baseline BP, intraoperative BP, and immediate postoperative BP. Future studies, including several ongoing trials, are expected to further address these questions.

A nonlinear relationship between SBP and stroke outcome after stroke onset was previously found by Shin et al. (2020). A nonlinear curve appeared immediately after stroke onset, and the relationship between SBP and poor outcome was U‐shaped. Subsequently, SBP and outcome exhibited a linear relationship from several hours to 1 day after stroke onset, and a nonlinear relationship was observed from 48 to 72 h. In this study, we proved that the relationship between SBP and functional outcome was overall linear except at 16 h after EVT. The reasons for this difference could be as follows. First, BP management for patients who received EVT was different from that for patients without reperfusion therapy. Second, due to the strict management of BP after EVT in our stroke center, the BP levels of most patients at different time points after EVT were controlled within a relatively stable and uniform range. We consider that this exactly reflects the rationality of post‐EVT BP management for LVO patients in our stroke center.

Furthermore, our study showed that patients with LVO stroke who received EVT had various trajectories, which were associated with functional outcome. We observed a significantly increasing risk of functional dependence in the high‐to‐low group, which was discrepant with previous findings (Petersen et al., 2022). The reason could be that patients who receive EVT and BP management differ among stroke centers. Previous findings found that patients with higher mean SBP after EVT had an increased risk of malignant brain edema, which was consistent with our result that SBP should be maintained at a relatively low level in the early stage after EVT (Anadani et al., 2020). The results of our study cannot conclusively answer when and how to intervene in BP after EVT, and specific targets for interventions need to be tested in further prospective clinical trials.

Our results have implications for clinical practice and may have a positive influence on the development of BP management and clinical trials for AIS. However, this study also contains some limitations. First, a retrospective observational design was used in the present study. Therefore, we could not conclude a causal relationship between BP and poor outcome based on the present results. Second, because BP data were extracted retrospectively from our electronic medical record systems and the 3‐month mRS was collected prospectively, a certain amount of BP and follow‐up data were missing. However, the missing data accounted for only 7.6% of the total data, which was within the normal range of loss to follow‐up, so it did not affect the results of our study. Nonetheless, until now, our study is the first to investigate the role of BP levels at different time points after EVT in patients with LVO stroke. Further RCTs and prospective studies are warranted.

## 5 CONCLUSIONS

In summary, higher SBP within the first 72 h after EVT has a time‐dependent association with adverse clinical outcomes. Our findings suggest that BP management strategy should consider the change of BP effect on outcome over time after EVT. Further studies are needed to identify the optimal BP at each time point after EVT.

## AUTHOR CONTRIBUTIONS

**Xin Jiang**: Writing—original draft; writing—review and editing; conceptualization; methodology; software; data curation; formal analysis; investigation. **Jinghuan Fang**: Conceptualization; investigation; writing—original draft; writing—review and editing; methodology; data curation; formal analysis; software. **Lijie Gao**: Conceptualization; methodology; software; formal analysis; writing—review and editing; writing—original draft. **Shuju Dong**: Conceptualization; methodology; writing—original draft; writing—review and editing. **Jian Wang**: Conceptualization; methodology; writing—review and editing; writing—original draft. **Fayun Hu**: Conceptualization; methodology; writing—review and editing; writing—original draft; supervision; data curation; investigation; formal analysis; resources; visualization. **Li He**: Conceptualization; methodology; writing—review and editing; writing—original draft; supervision; project administration; data curation; investigation; formal analysis; visualization; resources.

## CONFLICT OF INTEREST STATEMENT

The authors declared no potential conflicts of interest with respect to the research, authorship, and/or publication of this article.

### INSTITUTIONAL REVIEW BOARD STATEMENT

This study was performed in accordance with the ethical principles of the 1964 Declaration of Helsinki and approved by the Ethics Committee of West China Hospital [No. 2020(69)].

### PEER REVIEW

The peer review history for this article is available at https://publons.com/publon/10.1002/brb3.3442.

## Supporting information

Supplemental material for this article is available online.

## Data Availability

The data that support the findings of this study are available from the corresponding author on reasonable request.
