# Supplementary material for: Time‐dependent systolic blood pressure within 72 h after endovascular treatment in large vessel occlusion stroke
Source: Brain Behav. 2024 Mar 7;14(3):e3442. doi: 10.1002/brb3.3442 (PMC10918593; doi:10.1002/brb3.3442)

**Supplemental material**

Supplemental Table S1 Characteristics of systolic and diastolic blood pressure at each time point.

|  | Overall | mRS 0-2 | mRS 3-6 | *P* |
| --- | --- | --- | --- | --- |
|  | N=406 | N=142 | N=264 |  |
| SBP 1-hour | 129.7 (22.4) | 127.3 (21.0) | 131.1 (23.0) | 0.108 |
| DBP 1-hour | 74.1 (12.9) | 73.2 (13.4) | 74.6 (12.5) | 0.294 |
| SBP 2-hour | 126.6 (19.4) | 123.8 (19.0) | 128.1 (19.6) | 0.038 |
| DBP 2-hour | 72.7 (12.1) | 72.2 (13.5) | 72.9 (11.3) | 0.608 |
| SBP 4-hour | 121.0 (17.7) | 119.0 (18.2) | 122.1 (17.4) | 0.098 |
| DBP 4-hour | 69.7 (11.5) | 69.6 (12.5) | 69.7 (10.9) | 0.896 |
| SBP 8-hour | 119.6 (16.7) | 118.0 (17.1) | 120.5 (16.5) | 0.147 |
| DBP 8-hour | 68.4 (11.8) | 68.4 (11.3) | 68.3 (12.0) | 0.952 |
| SBP 16-hour | 118.8 (15.4) | 117.8 (16.4) | 119.4 (14.7) | 0.327 |
| DBP 16-hour | 68.3 (11.0) | 68.1 (11.7) | 68.5 (10.6) | 0.733 |
| SBP 24-hour | 120.7 (16.6) | 119.1 (15.1) | 121.6 (17.4) | 0.151 |
| DBP 24-hour | 69.0 (11.6) | 69.3 (11.5) | 68.8 (11.7) | 0.709 |
| SBP 48-hour | 122.0 (17.2) | 119.5 (16.2) | 123.6 (17.6) | 0.027 |
| DBP 48-hour | 70.1 (12.3) | 69.3 (11.6) | 70.6 (12.6) | 0.307 |
| SBP 72-hour | 122.4 (17.7) | 119.5 (16.5) | 124.3 (18.2) | 0.014 |
| DBP 72-hour | 70.3 (12.4) | 70.1 (12.1) | 70.5 (12.6) | 0.759 |

Supplemental Table S2 SBP Trajectories and Clinical Outcomes.

|  | Non-adjusted | | Adjusted | |
| --- | --- | --- | --- | --- |
|  | OR | P value | OR | P value |
| Moderate | ref | | ref | |
| Low-to-high | 1.62 (0.69, 3.82) | 0.2683 | 0.92 (0.31, 2.72) | 0.8826 |
| High-to-low | 3.19 (1.50, 6.79) | 0.0026 | 3.88 (1.67, 9.02) | 0.0016 |

Supplemental Figure S1 Distributions of SBP at each time-point


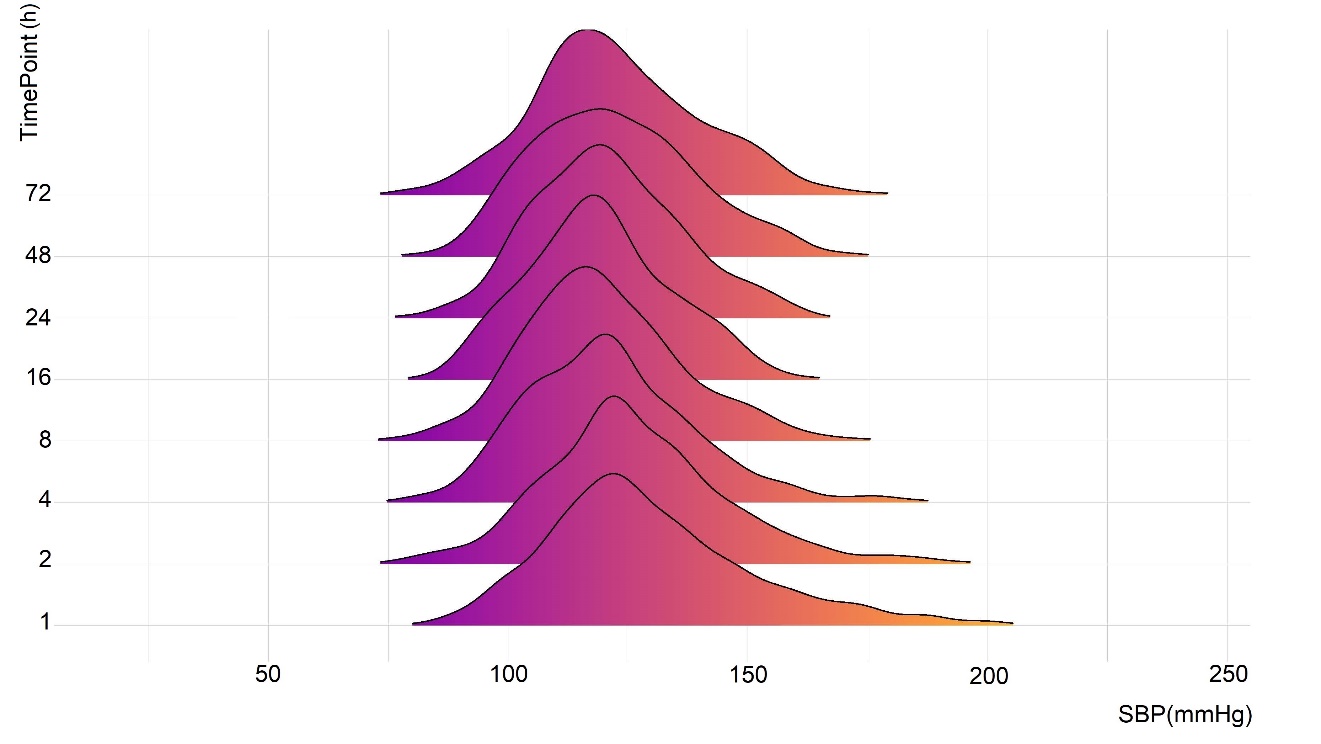


Supplemental Figure S2 Distributions of DBP at each time-point.


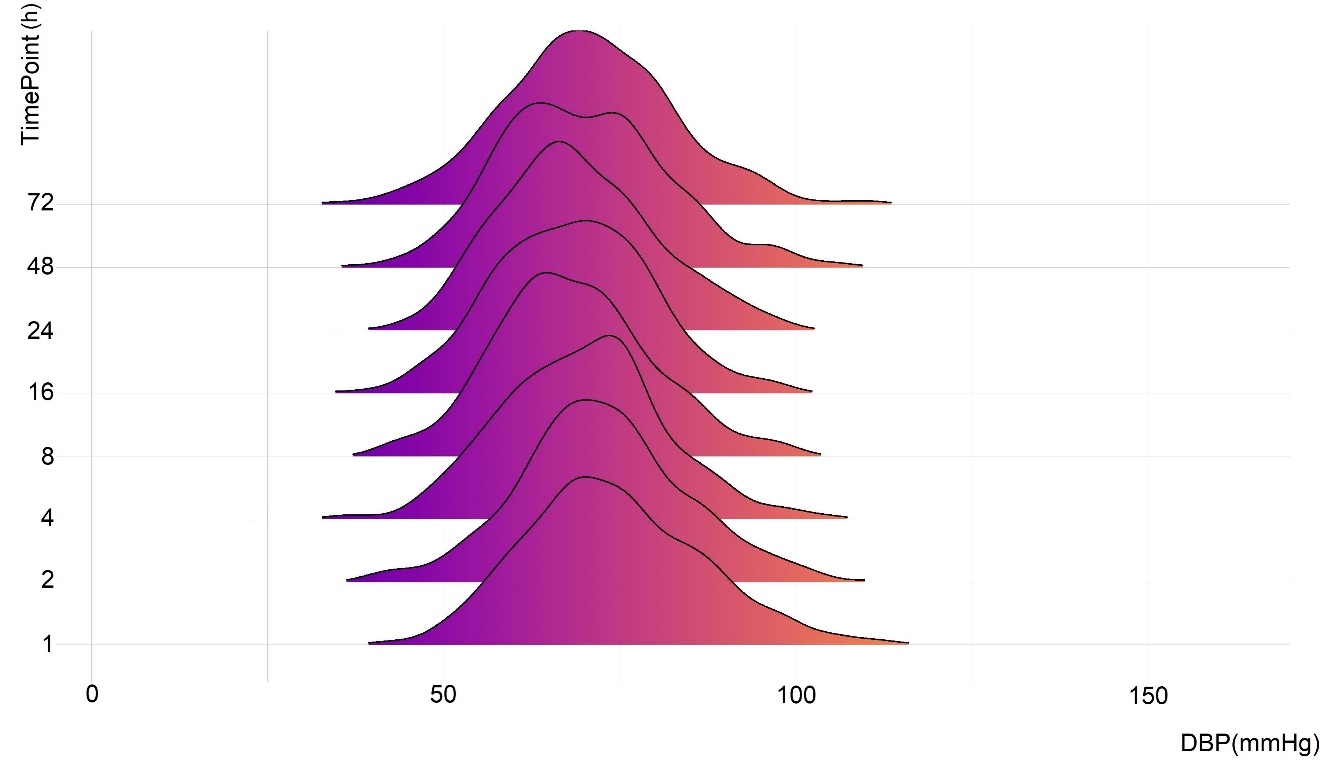


Supplemental Figure S3 Distributions of functional dependence (3-month mRS 3-6) according to SBP at each time-point.


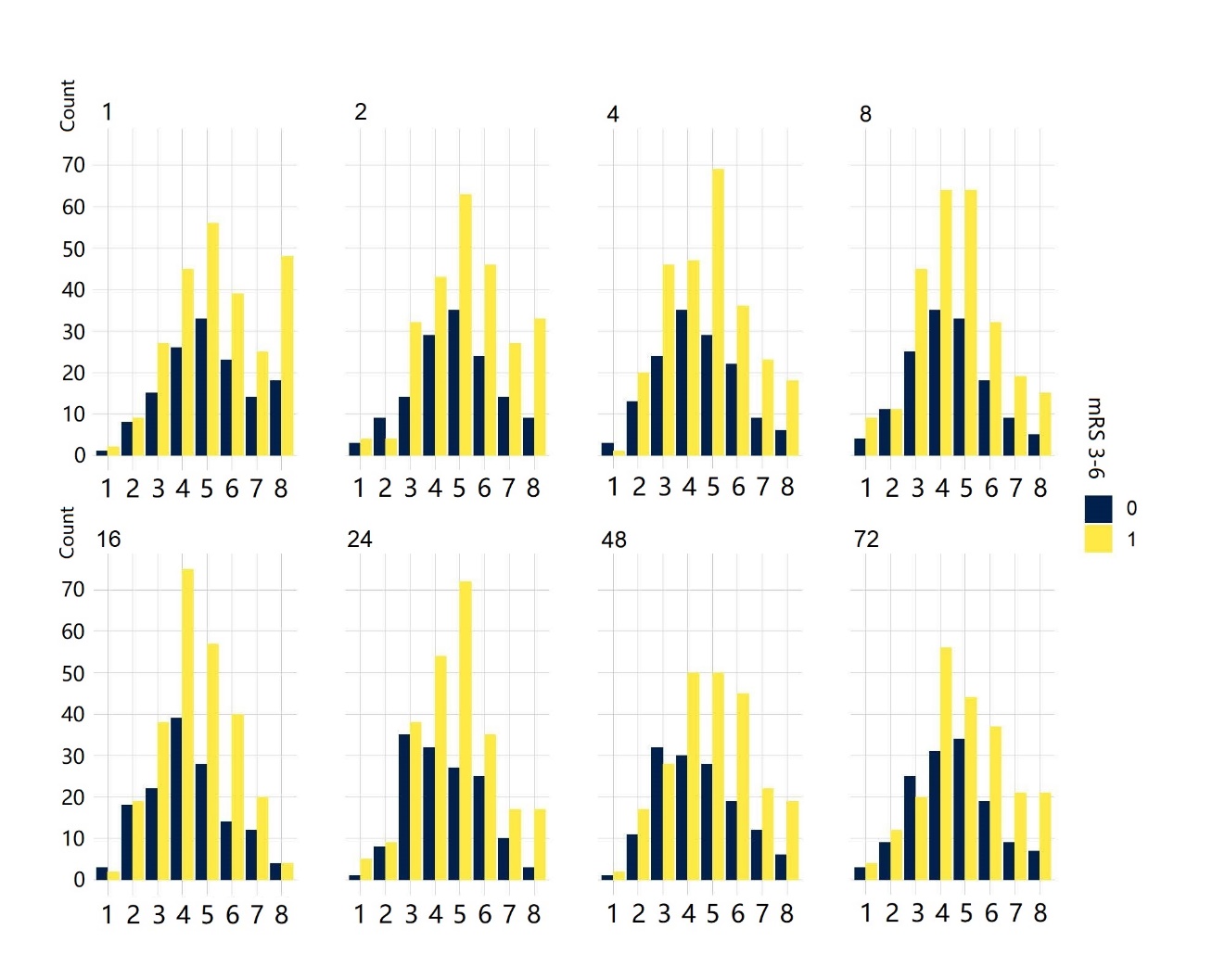


Footnotes:

1: ˂90 mmHg, 2: 90-100 mmHg; 3: 100-110 mmHg; 4: 110-120 mmHg; 5:120-130 mmHg; 6: 130-140 mmHg; 7: 140-150 mmHg; 8: ˃150 mmHg

Supplemental Figure S4 Distributions of functional dependence (3-month mRS 3-6) according to DBP at each time-point.


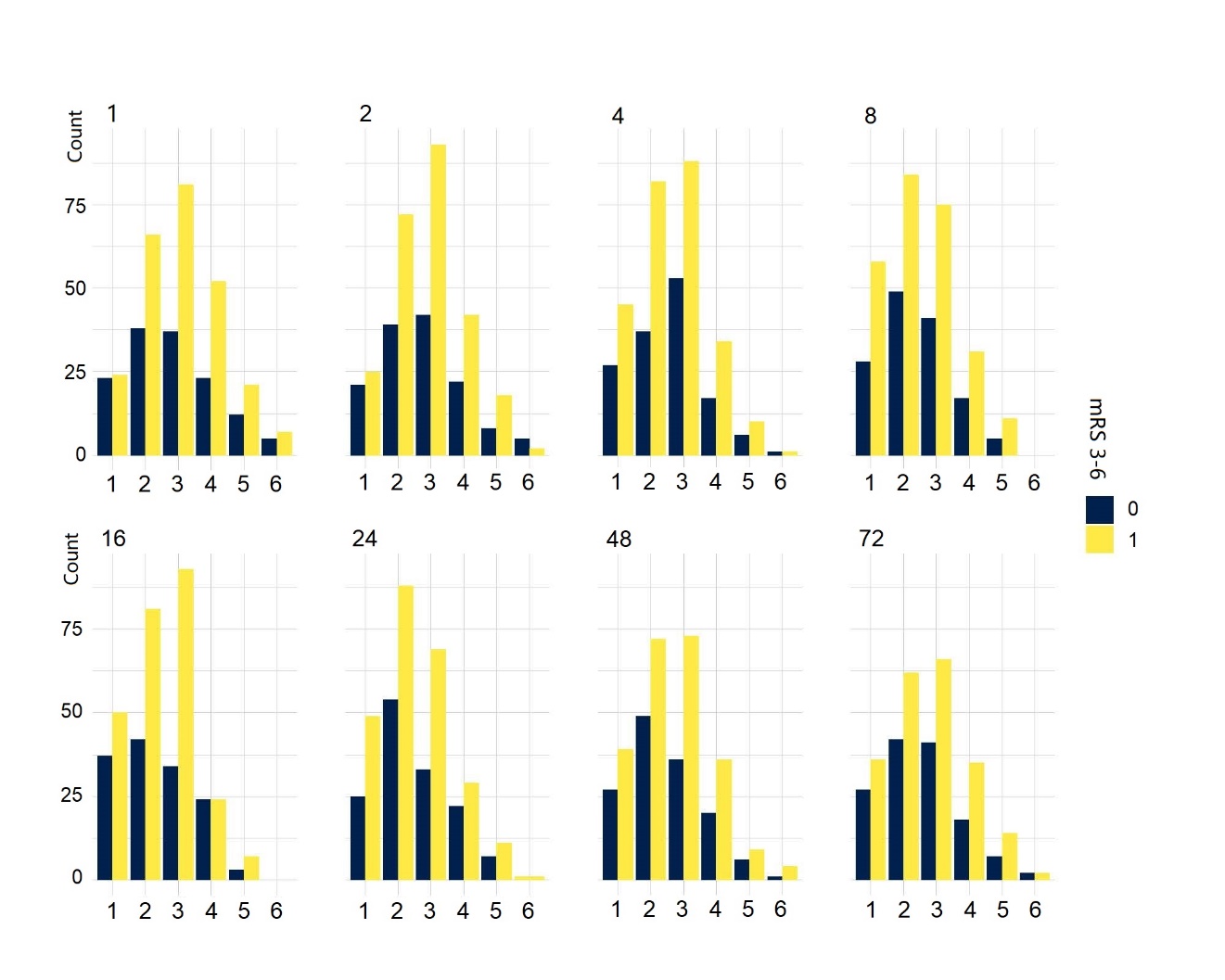


Footnotes:

1: ˂60 mmHg, 2: 60-70 mmHg; 3: 70-80 mmHg; 4: 80-90 mmHg; 5: 90-100 mmHg; 6: ˃100 mmHg

Supplemental Figure S5 Probability of functional dependence (3-month mRS 3-6) predicted by DBP at each time-point.


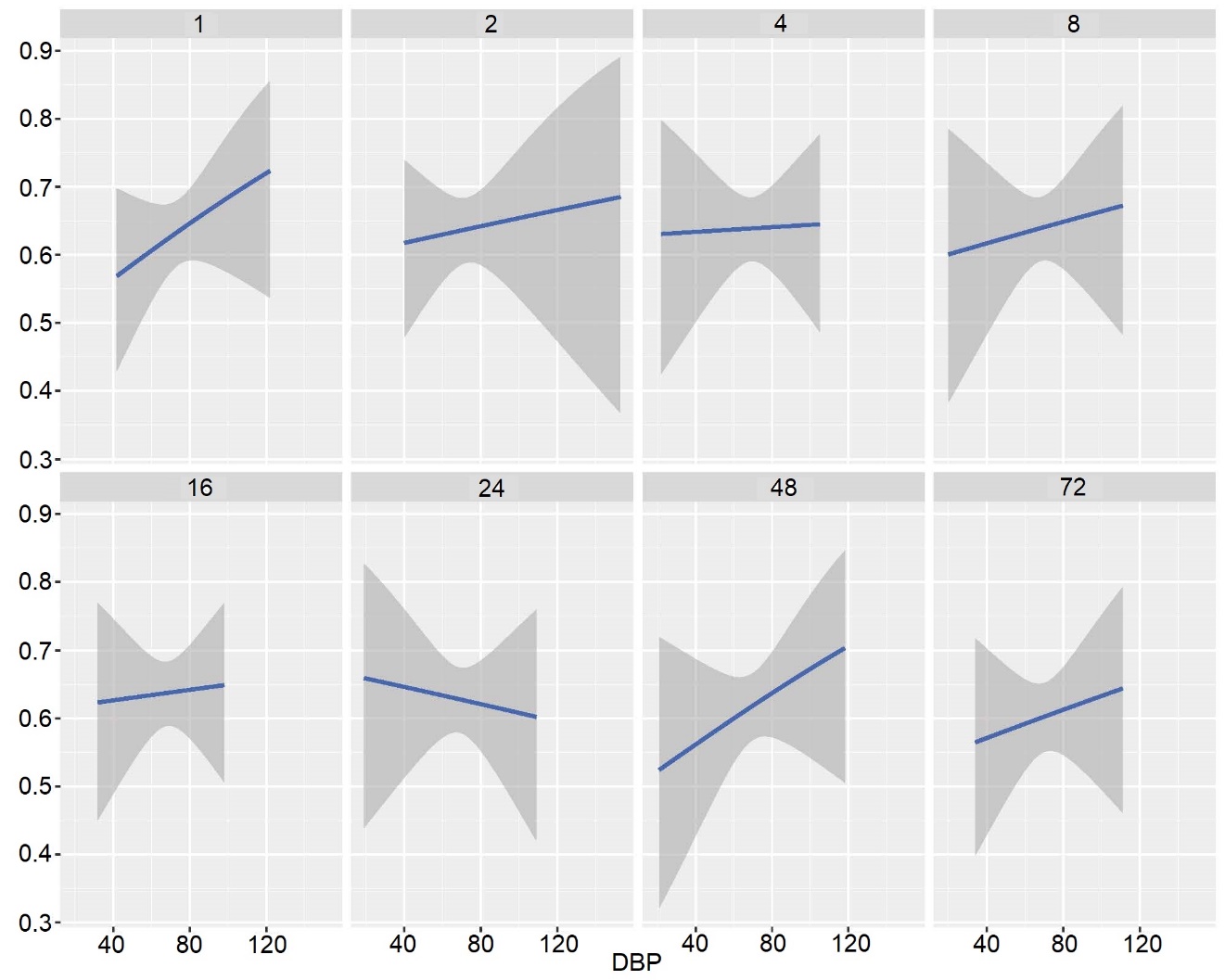

Supplement: Supplementary file 1 — Supplemental material for this article is available online. [file BRB3-14-e3442-s001.docx]
